# Transcriptomic responses under combined bacterial blight and drought stress in rice reveal potential genes to improve multi-stress tolerance

**DOI:** 10.1186/s12870-022-03725-3

**Published:** 2022-07-18

**Authors:** Garima Pal, Rahul Bakade, Sanjay Deshpande, V. Sureshkumar, Swathi S. Patil, Akashata Dawane, Subham Agarwal, Vidya Niranjan, M. K. PrasannaKumar, Ramu S. Vemanna

**Affiliations:** 1 Laboratory of Plant Functional Genomics, Regional Centre for Biotechnology, Faridabad-Gurgaon Expressway, NCR Biotech Science Cluster, 3rd Milestone, Faridabad, Haryana 121 001 India; 2 Department of Plant Pathology, University of Agricultural Sciences, GKVK, Bengaluru, 560065 India; 3 Department of Biotechnology, R.V. Engineering College, Bengaluru, 560059 India

**Keywords:** Drought, Xanthomonas, Rice, Combined stress, Transcriptome, Meta-analysis, Translation, Climate change, Transcription factor, Kinases

## Abstract

**Background:**

The unprecedented drought and frequent occurrence of pathogen infection in rice is becoming more due to climate change. Simultaneous occurrence of stresses lead to more crop loss. To cope up multiple stresses, the durable resistant cultivars needs to be developed, by identifying relevant genes from combined biotic and abiotic stress exposed plants.

**Results:**

We studied the effect of drought stress, bacterial leaf blight disease causing *Xanthomonas oryzae* pv. *oryzae (Xoo)* pathogen infection and combined stress in contrasting BPT5204 and TN1 rice genotypes. Mild drought stress increased *Xoo* infection irrespective of the genotype. To identify relevant genes that could be used to develop multi-stress tolerant rice, RNA sequencing from individual drought, pathogen and combined stresses in contrasting genotypes has been developed. Many important genes are identified from resistant genotype and diverse group of genes are differentially expressed in contrasting genotypes under combined stress. Further, a meta-analysis from individual drought and *Xoo* pathogen stress from public domain data sets narrowed- down candidate differentially expressed genes. Many translation associated genes are differentially expressed suggesting their extra-ribosomal function in multi-stress adaptation. Overexpression of many of these genes showed their relevance in improving stress tolerance in rice by different scientific groups. In combined stress, many downregulated genes also showed their relevance in stress adaptation when they were over-expressed.

**Conclusions:**

Our study identifies many important genes, which can be used as molecular markers and targets for genetic manipulation to develop durable resistant rice cultivars. Strategies should be developed to activate downregulated genes, to improve multi-stress tolerance in plants.

**Supplementary Information:**

The online version contains supplementary material available at 10.1186/s12870-022-03725-3.

## Introduction

Plants are sessile, exposed to diverse biotic and abiotic stresses leads to reduction in yields of many agricultural economically important crops [1]. Rice is one of the most important staple food which feeds more than half of the population globally. Due to climate change, the frequency of uneven rainfall and severe drought stresses are common, which threaten the crop production [2]. During drastically changing climatic conditions, many bacterial pathogens can infect plants and reduce the yield. Rice being grown in the puddled condition is more sensitive to uneven rainfall and drought stress. Besides its direct effect on the crop, drought stress alters plant-pathogen interaction and disease development [3]. The occurrence and severity of combined biotic and abiotic stresses, depend on host resistance or susceptibility, duration of stress exposure and pathogen race [4]. Evidences suggest that, plant responses overlap for drought and bacterial stresses in many crops like *Arabidopsis*, rice, chickpea, sunflower and several cross-talk mechanisms have been identified [1, 5–7]. Transcriptomic and meta-analysis approaches using expression profile between biotic and abiotic stresses have revealed unique genes which perform similarly across different stress stimuli [8, 9]. Interestingly phytohormone cross-talk mechanisms share many common responsive genes in combined multiple stresses [10–12].

Multiple QTLs for drought resistance and resistance against bacterial blight caused by *Xanthomonas oryzae* pv. *oryzae (Xoo)* bacteria has been identified [3, 13]. The introgression of *Xa21*, *Xa5, Xa13* conferred broad spectrum resistance in different rice cultivars against bacterial infection [14]. Studies have shown genotype dependent pathogen infection in rice under drought-induced conditions [3]. Genotypes with suitable *Xa* genes provide resistance against bacterial blight under drought conditions. The introgression of R genes *Xa4* and *Xa7* in near isogenic lines confers resistance against bacterial blight under high temperature [15]. Combined stress of high temperature and bacterial blight, drought stress and bacterial blight at seedling stage found multiple *Xa* genes, which can be introgressed to improve resistance [7]. Combined stress tolerance was improved by the introgression of four resistance genes (*Xa4, xa5, xa13* and *Xa21)* with submergence (*Sub1*), salinity (*Saltol*), blast (*Pi2*, *Pi9*) and gall midge (*Gm1*, *Gm4*) [16]. Evidences suggest that, introgression of multiple drought QTLs along with many R genes in an elite genotype can provide tolerance against combined stress in rice [3].

Multiple stress tolerance is governed by several genes, to develop durable resistant genotypes, evaluating rice varieties under combined stresses is the best strategy [3]. The candidate genes which are involved in multi-stress tolerance may be identified in plants exposed to combined stress. Existing reports suggested that, several overlapping genes in *Xoo* and drought stress play role in improving tolerance. Transcriptome data of drought and *Xoo* infection showed 2276 overlapping genes which were differentially expressed [8]. Meta-analysis study of transcriptome from drought and bacterial blight combined stress, 5084 and 1618 differentially expressed genes (DEGs) were identified in rice and Arabidopsis respectively [17]. Meta-analysis of sunflower transcriptome revealed 526 upregulated and 4440 downregulated genes in combined stress of drought and pathogen along with NaCl, cold and oxidative stress [5]. These studies identified the genes by comparing the individual stress transcriptome data. Comparative study for drought and *Xoo*, in resistance rice line H471 and its recurrent parent HHZ identified 306 and 840 DEGs, and 178 genes were common among both stresses [18].

We made an attempt to identify candidate genes in rice plants exposed to the combined stress of drought and bacterial blight causing pathogen infection. The major challenge in studying the multiple stresses, is imposition of combined stresses simultaneously [19]. Severe drought stress reduces the bacterial multiplication due to higher leaf water loss [20]. To overcome this, we optimized a combined stress imposition method in rice by gradually reducing the soil moisture content and subsequently infecting pathogen. A comparative transcriptomic data from contrasting BPT5204 and TN1 rice genotype was developed. Several relevant genes for individual and combined stress, regulating different pathways were identified. A meta-analysis from individual drought and *Xoo* infected rice was performed, using public microarray datasets. Several DEGs identified were characterized in stresses for either abiotic or biotic factors. Our results demonstrated that several genes are involved in multi-stress tolerance. The identified genes can be used as genetic markers and candidate genes for crop improvement programs.

## Results

### Differential response of contrasting rice genotypes to combined drought stress and pathogen infection

**Fig. 1:**
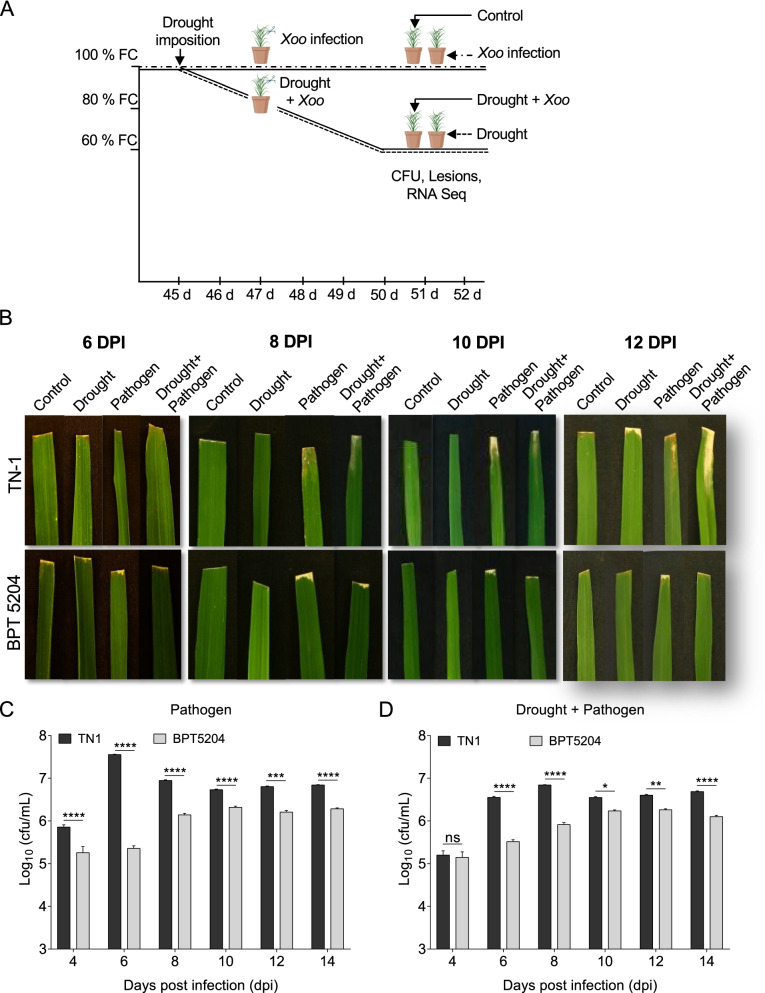
Combined stress response of contrasting rice genotypes. **A** Scheme showing combined and individual drought and pathogen stress imposition method. Drought stress was imposed to 45-days-old plants by gradual reduction in moisture content and maintained upto 60% FC. *Xoo* was infected to 47-days old plants at 80% FC with 0.5 × 10^8^ CFU/mL using leaf-clipping method. For combined stress, when plants reached 80% FC, infected with *Xoo* and maintained upto 60% FC. **B** Bacterial disease symptoms in BPT5204 and TN1 plants exposed to drought, pathogen and combined stress at 6, 8, 10 and 12 dpi. **C** Bacterial multiplication rate from 4 to 14 days in contrasting rice genotypes under pathogen stress. **D** Bacterial multiplication rate in combined stress were measured from 4 to 14 days. Minimum five biological replicates were maintained for each stress. Graphs showing mean values ± SE. Significant differences were determined at *p* < 0.0001 with one-way ANOVA using Tukey’s HSD analysis

To study the responses of rice under drought, pathogen and combined stress, two contrasting BPT5204 and TN1 genotypes were maintained in four different sets. One set of 45-days-old plants were infected with *Xoo* and another set was exposed to drought stress by gradually reducing soil moisture content upto 60% field capacity (FC). For combined stress (*Xoo* and drought stress), the 45-days-old plants were exposed to drought stress by reducing FC to 80% for two days and infected with 0.5 × 10^8^ CFU/mL of concentration of *Xoo* by leaf clipping method. Further, moisture level was reduced to 60% FC and plants were maintained for four days (Fig. 1A). Disease pattern and bacterial multiplication rates were assessed in resistant BPT5204 and susceptible TN1 genotypes. After 4 dpi, *Xoo* pathogen infection rate and lesions were measured at different time intervals. The susceptible TN1 genotype showed higher infection under drought condition (Fig. 1B) at 6 dpi and progressed severely till 12 dpi, whereas, in BPT5204 bacterial infection progression was slow. In case of combined stress, TN1 genotype showed higher susceptibility and even BPT5204 showed higher lesions compared to individual pathogen infection. TN1 genotype showed > 1-2 fold higher pathogen multiplication than BPT5204 at 4, 6, 8, 10, 12 and 14 dpi, whereas at 6 dpi > 2 fold pathogen multiplication was observed in only pathogen infected plants. Increased lesions were observed in TN1, whereas, BPT5204 maintained less bacterial growth as well as disease symptoms (Fig. 1B and C). In case of combined stress, drought stress prior to pathogen infection resulted in reduced bacterial multiplication in both genotypes (Fig. 1D). At 4 dpi, no significant difference was observed in bacterial multiplication rate in both the genotypes, whereas at 6, 8, 10, 12 and 14 dpi TN1 showed higher bacterial multiplication compared to BPT5204.

**Fig. 2:**
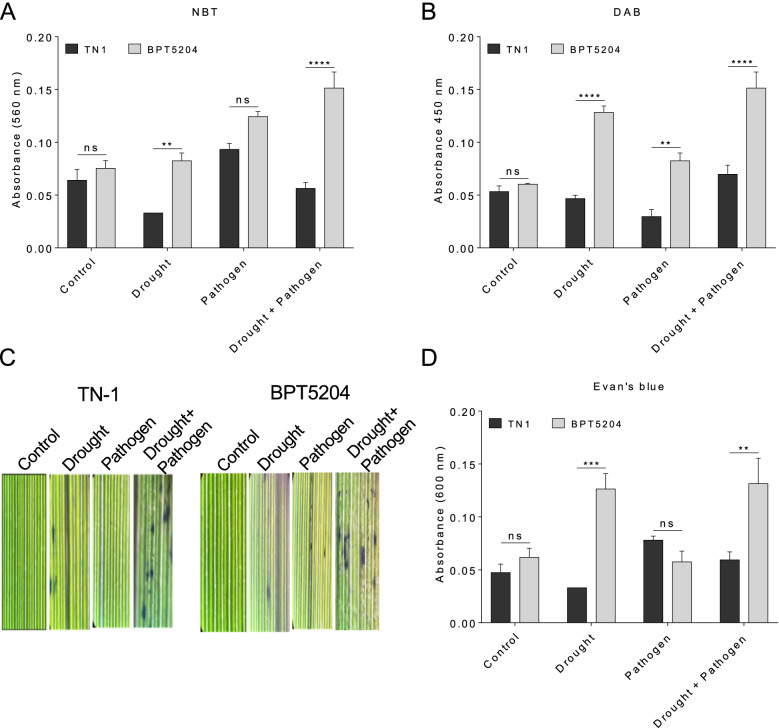
Effect of individual and combined stress response on rice plants. **A** Level of superoxide radicles in drought, pathogen and combined stress. After two days of plants reaching to severe stress, leaves were stained with NBT. **B** Levels of H_2_O_2_ in drought, pathogen and combined stress was quantified using DAB staining. **C** Photographs showing Evan’s blue staining to measure membrane stability. **D** Quantification of Evan’s blue dye accumulation. Minimum five biological replicates were used for quantification. Graphs showing mean values ± SE. Significant differences were determined at *p* < 0.0001 (estimated by one-way ANOVA using Tukey’s HSD analysis

The individual and combined stress effect were quantified, by measuring reactive oxygen species (ROS) such as superoxide and H_2_O_2_. Superoxide estimation using NBT staining showed higher level of ROS accumulation in combined as well as drought stress. In drought stress > 2.5 fold levels of superoxide was accumulated in BPT5204, whereas, in pathogen infection there was no significant difference was observed in both contrasting genotypes. Similarly, in combined stress > 2.5 fold accumulation was observed in BPT5204 compared to TN1 (Fig. 2A). H_2_O_2_ quantification using DAB was observed > 2.5 fold in BPT5204 in individual stress whereas in combined stress accumulation was ~ 10 fold higher compared to TN1 (Fig. 2B). The effect of stress on cell membrane was quantified using Evan’s blue in individual as well as combined stress (Fig. 2C). In drought stress, membrane damage was observed > 3.5 fold in BPT5204 compared to TN1 whereas, in pathogen infection there was no significant difference was observed. In combined stress, accumulation of Evan’s blue dye was > 2 fold in BPT5204 compared to TN1 genotype (Fig. 2D).

### Transcriptional profiling identifies common and unique genes in combined stress

**Fig. 3:**
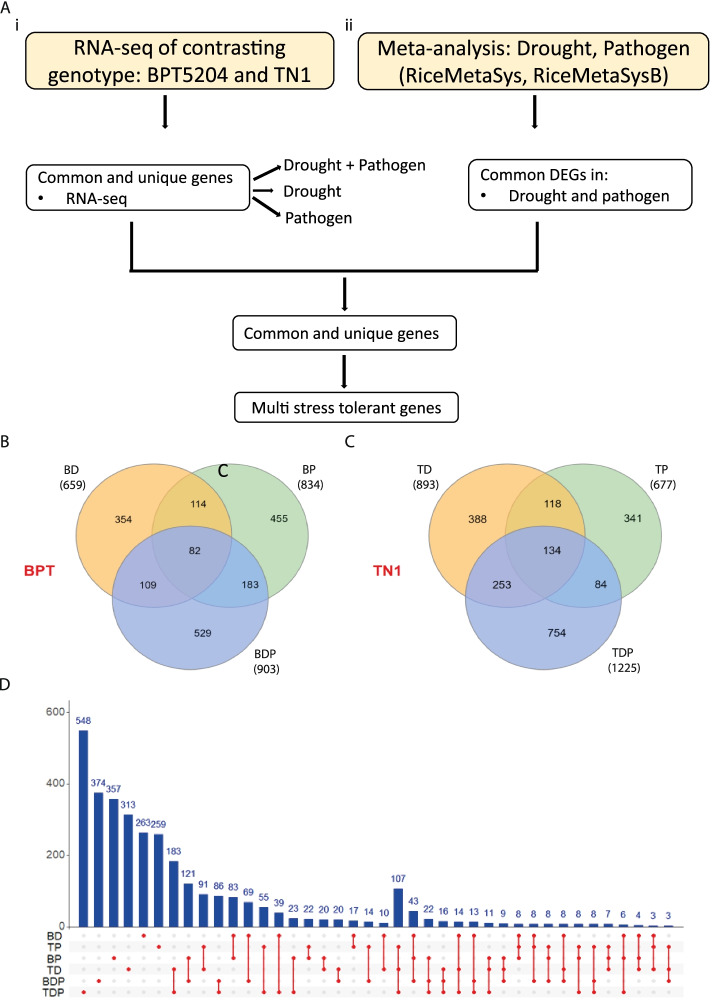
Differentially expressed genes under combined and individual *Xoo* and drought stress. **A** Scheme showing strategy followed to identify multi-stress tolerant genes, (i) RNA sequencing data developed from this study to identify common and unique genes, (ii) Meta-analysis data from RiceMetasysA http://14.139.229.201/RiceMetaSys/ and RiceMetasysB http://14.139.229.201/RiceMetaSysB/. **B** Venn diagram showing differentially expressed genes in combined, pathogen and drought stress in resistant BPT5204 genotype, BD-drought, BP -pathogen and BDP- Drought + pathogen, and **C** Sensitive TN1 genotype, TD-drought, TP -pathogen and TDP- Drought + pathogen, **D** Differentially expressed genes in BPT5204 and TN1 in drought, pathogen and combined stress

The emphasis of the study is to identify the candidate genes which can help in improving the combined stress tolerance in rice. We followed two approaches, initially RNA sequencing data was developed from contrasting rice genotypes that are exposed to individual and combined stress to identify common and unique genes (Fig. 3A i, Additional file 1). In another approach, microarray data from individual drought and pathogen stress from public domain was analysed (Additional file 1) and common differentially expressed genes (DEGs) were identified (Fig. 3A ii).

The transcriptomic data from four different sets i.e. control, drought, pathogen and combined stress for both BPT5204 and TN1 genotypes were developed. Around 98.16% of the high-quality reads were mapped to the reference genome. A total of 3381 unique DEGs were identified across the stress treatments (Additional file 1). We identified 903 (BDP-Combined stress), 659 (BD-Drought), and 834 (BP-Pathogen) DEGs in BPT5204. In TN1 genotype, 1226 (TDP-Combined Stress), 893 (TD-Drought), and 677 (TP-Pathogen) DEGs were observed (Additional file 1). In the BPT5204, 191 genes from 903 DEGs in combined stress were found to be common in drought stress, and 265 DEGs were common in pathogen infection (Fig. 3B). Similarly, in TN1, 387 and 218 of DEGs from combined stress were found to be common with drought stress and pathogen infection respectively (Fig. 3C). In all treatments, 82 and 134 genes were expressed in BPT5204 and TN1 genotypes, respectively. The transcriptomic data revealed that in BPT5204, 374 genes and in TN1, 548 genes were uniquely expressed in combined stress (Fig. 3D, Additional file 1).

**Fig. 4:**
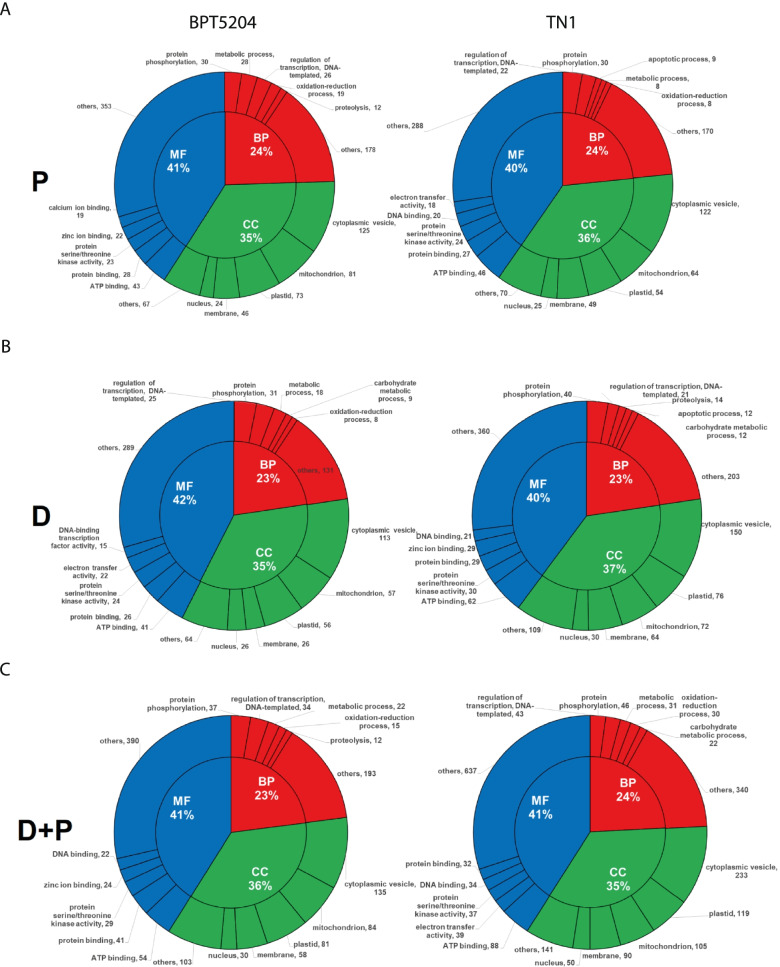
Classification of differentially expressed genes. Differentially expressed genes from BPT5204 and TN1 were characterized based on molecular function, cellular components and biological processes under (**A**) Pathogen stress -P (**B**) Drought stress -D (**C**) Combined drought and pathogen (D + P) stress. GO analysis was performed using agriGO (https://agrigo.rw/)

Gene ontology analysis was performed to classify genes in different categories. In pathogen infection, BPT5204 and TN1 genotypes showed more number of genes in molecular function (41% and 40% respectively) followed by cellular components (35% and 36% respectively) and biological processes (24% both) (Fig. 4A, Additional file 2). Likewise in drought stress, DEGs from both BPT5204 and TN1 genotypes represented more number of genes in molecular function (42% and 40% respectively) followed by cellular components (35% and 37% respectively) and biological processes (23% both) (Fig. 4B, Additional file 2). In combined stress, BPT5204 genotype represented 41% of genes in molecular function having ATP binding, protein binding, kinase activity, zinc binding and DNA binding activity. In TN1 genotype, 41% of genes represented in molecular functions of ATP binding, electron transfer, kinase activity, DNA binding and protein binding groups. In BPT5204 and TN1, 36% and 35% of genes respectively were represented in cellular components belonging to cytoplasmic vesicle, mitochondrion, plastid, membrane, nucleus and others. 23% of genes in BPT5204 and 24% of genes in TN1 were represented in biological processes belonging to protein phosphorylation, regulation of transcription, metabolic process, oxidation–reduction process, proteolysis and others (Fig. 4C, Additional file 2).

**Fig. 5:**
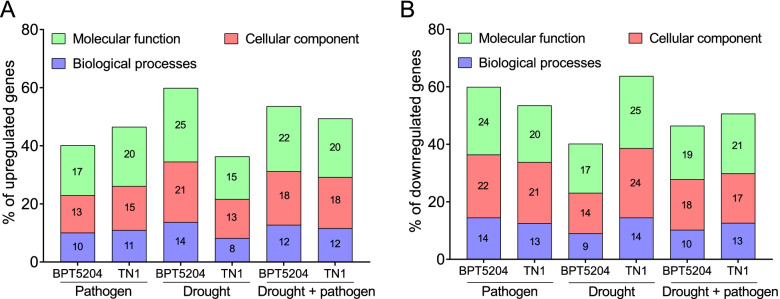
Number of upregulated and downregulated genes under combined and individual drought and pathogen stress. **A** Percentage of upregulated genes in molecular function, cellular components and biological processes under pathogen, drought and combined stress. **B** Percentage of downregulated genes in molecular function, cellular components and biological processes under pathogen, drought and combined stress

The upregulated genes in combined stress, 22% of genes were represented in molecular function in BPT5204 genotype and 20% of genes in TN1 (Fig. 5A, Additional file 3). In downregulated genes, upon combined stress more number of genes were downregulated in TN1 belonging to molecular function (21%), whereas in BPT5204, 19% of genes were represented (Fig. 5B). Several common genes from both the genotypes were identified. To assess the role of these genes, a PubMed search was conducted to know their relevance in plant stress adaptation. Interestingly, the role of many genes in plants were validated for different stresses by many research groups across the globe (Table 1).

**Table 1:** List of upregulated and downregulated genes in combined stresses. Literature survey showing annotation from different studies. Data was curated manually

Annotation		Functional relevance studies	Reference
**Upregulated in BPT5204 all stresses**			
Os01g0106400	Similar to Isoflavone reductase homolog IRL (EC 1.3.1.)	Oxidoreductase activity; Overexpression leads to tolerance to ROS in rice	Kim et al., 2009
Os01g0162500	Leucine-rich repeat-containing N-terminal, protein	Stress (Cold and drought) tolerance in rice; Required for Xa-21 transcript expression and Xa-21 mediated immunity in rice against Xoo	Liao et al.; 2016; 2. Caddell et al., 2017
Os01g0844300	Peptidylprolyl isomerase	Aids in protein folding	Shaw, 2002
Os02g0178800	Glossy1(GL1) homolog	Cuticular wax biosynthesis, Drought resistance in rice	Islam et al., 2009
Os02g0323000	Felis catus multi-drug resistance related (Fragment)	ATPase activity, ATPase-coupled transmembrane transporter activity and ATP binding	UNIPROT
Os02g0820200	Serine/threonine protein kinase	Involved in abiotic stress (salt and osmotic) tolerance in *Arabidiopsis*	Zang et al., 2016
Os03g0140400	Cytochrome P450 protein CYP96B4	Involved in growth regulation and drought stress response in rice	Tamiru et al., 2015
Os04g0266900	Transketolase C-terminal-like domain containing protein	Acts as a regulatory molecule binding site	Pfam, InterPro
Os06g0544100	Tyrosine protein kinase domain containing protein	Abiotic stress (drought, heat, salt and sumergence) tolerance	Elangovan et al., 2020
Os07g0194100	OSK2	Play important role in the early stages of endosperm development in rice seeds	Takano et al., 1998
Os07g0674400	Pollen Ole e 1 allergen/extensin domain containing protein	Pollen germination and pollen tube growth	Jimenez-Lopez et al., 2012
Os09g0472900	Blight-associated protein p12 precursor	p12 is responsinle for systemic acquired resistance in citrus plants against citrus blight	Uniprot
Os12g0133300	Carbohydrate transporter/ sugar porter/ transporter	Involved in long distance transport of sucrose from source to sink where sugars are used or stored	Doidy et al., 2012
Os12g0454800	Receptor-like serine/threonine kinase	Cytokinin signaling	Afzal et al., 2008
**Upregulated in TN1 all stresses**			
Os01g0858000	WD40 repeat domain containing protein	Function as molecular hubs mediating supramolecular interactions, involved in plant cell wall biosynthesis; Histone modification, transcription regulation and signal transduction	Guerriera et al., 2015; Hu et al., 2018
Os02g0536300	Leucine-rich repeat 2 domain containing protein	Initiate immune response in plants by sensing PAMP	Ng and Xavier., 2011
Os02g0599151	Probable protein phosphatase 2C 17	Regulate various plant signal transduction pathways (MAPK signalling and ABA signalling)	Rodriguez et al., 1998; Endo et al., 2018
Os03g0188100	transparent testa 12 protein	encodes a multidrug secondary transporter-like protein required for flavonoid sequestration in vacuoles of the seed coat endothelium in *Arabidopsis*	Debeaujon et al., 2001
Os03g0305800	Galactosyl transferase family protein	Biosynthesis of plant cell wall, synthesis of diverse secondary metabolites and modification of plant hormones	Cao et al., 2008
Os03g0332900	Protein kinase, core domain containing protein	Central components in plant responses to environmental stresses such as drought, high salinity, cold, and pathogen attack	Wang et al., 2020
Os03g0448500	Calcineurin B-like protein	Decoding Ca2 + signatures elicited by a variety of abiotic stresses	Cho et al., 2016
Os03g0575200	Potassium transporter 1 (OsHAK1)	Positively regulates drought stress responses in rice	Chen et al., 2017
Os03g0785900	Similar to Glutathione-S-transferase	Involved in abiotic stress response and heavy metal (Arsenic) detoxification	Kumar and Trivedi (2018)
Os03g0820500	Similar to WCOR719	Involved in dynamic reorganisation of cytoskeleton during low temperature acclimatisation	Danyluk et al., 1996
Os04g0127500	Serine/threonine protein kinase-related domain containing protein	Involved in abiotic stress (salt and osmotic) tolerance in *Arabidiopsis*	Zang et al., 2016
Os04g0416100	Transcription factor E2F1 (E2F-1)	Expressed throughout the cell cycle, Elevates CDK levels and activity, even under hormone-free conditions	TAIR (The Arabidopsis Information Resource)
Os04g0584300	Similar to Catalytic/ protein phosphatase type 2C	Regulate various plant signal transduction pathways (MAPK signalling and ABA signalling)	Rodriguez et al., 1998; Endo et al., 2018
Os05g0576800	Blast and wounding induced mitogen-activated protein kinase	Activate MAPK pathway	Cheong et al., 2003
Os06g0191900	Serine threonine kinase	Act as receptors for external factors like environmental conditions and mediate cellular responses	Afzal et al., 2008
Os06g0229000	FtsH protease (VAR2) (Zinc dependent protease)	Involved with abiotic (light) stress response in Arabidopsis	Lopes et al., 2018
Os06g0275000	Zinc finger protein, Heading date	Involved in abiotic stress tolerance	Jin et al., 2018
Os06g0681200	Cupredoxin domain containing protein	Involved in abiotic stress responses	Jangam et al., 2016
Os06g0702100	Methyl-CpG DNA binding domain containing protein	Involved in DNA methylation and abiotic stress responses	Parida et al., 2018
Os07g0175600	Plant lipid transfer protein and hydrophobic protein,	Act as defense proteins in plant innate immunity, bind and transfer lipids and constitute one of the most clinically important classes of plant allergens	Finkina et al., 2016
Os07g0521500	NB-ARC domain containing protein	Regulate the activity of R protein	Ooijen et al., 2008
Os07g0614300	Von Willebrand factor type A domain containing protein,	Component of C-terminus of *Lagging Growth and Development 1 (LGD1)* and is responsible for the nuclear targeting and RNA binding activity	Thangasamy et al., 2012
Os10g0471000	Protein of unknown function DUF810	Involved in tolerance to drought and salt stress	Li et al., 2018
Os10g0521000	TRE1 protein (Fragment)	Required for glycogen metabolism	Zhang et al., 2021
Os10g0529700	glutathione transferase35	Involved in abiotic stress response and heavy metal (Arsenic) detoxification	Kumar and Trivedi, 2018
Os11g0201400	Histone deacetylase	Negatively regulates plant innate immunity by modulating histone H4 acetylation of defense-related genes in rice	Ding et al., 2012
Os11g0693800	ATP-citrate synthase	Negatively regulates plant innate immunity in rice	Ruan et al., 2018
Os12g0512100	Sugar/inositol transporter domain containing protein	Involved in transport of sugar from source to sink	Kong et al., 2019
**Downregulated in BPT in all stresses**			
Os02g0226200	HAD-superfamily subfamily IB hydrolase, hypothetical 1 protein	Involved in intracellular or extracellular organic phosphorous recycling under inorganic phosphorous stress conditions in plants	Du et al., 2021
Os02g0249300	Zinc finger, RING/FYVE/PHD-type domain containing protein	Involved in abiotic stress (salt and osmotic) tolerance in *Arabidiopsis*	Zang et al., 2016
Os03g0181750	ABC transporter, transmembrane domain domain containing protein	Required for normal plant development, detoxification and pathogen defense via the transport of xenobiotics and secondary metabolites across the plants	Hwang et al., 2016
Os04g0364800	Barwin-related endoglucanase domain containing protein	Aids in polysaccharide binding and Bawin is a putative plant defense protein; 2. All PR-4 proteins have Barwin domain	Todd et al., 2002; Franco et al., 2019
Os04g0385600	Tetratricopeptide-like helical domain containing protein	involved in plant stress (osmotic stress) and hormone signalling (abscisic acid)	Schapire et al., 2006; Sharma and Pandey, 2016
Os04g0618700	Protein kinase, core domain containing protein	Central components in plant responses to environmental stresses such as drought, high salinity, cold, and pathogen attack	Wang et al., 2020
Os04g0686000	Zinc finger, RING/FYVE/PHD-type domain containing protein	Involved in abiotic stress (salt and osmotic) tolerance in *Arabidiopsis*	Zang et al., 2016
Os05g0170600	CLE family OsCLE503 protein	Regulate cell proliferation and differentiation in plant shoots, roots, vasculature, and other tissues	Li et al., 2019
Os07g0162700	Alpha/beta hydrolase fold-3 domain containing protein	Associated with housekeeping roles that participate in the breakdown and recycling of cellular metabolites, processing of external nutrients and detoxification of xenobiotics	Gershater and Edwards, 2007
Os08g0113000	Peroxidase 47 precursor (EC 1.11.1.7) (Atperox P47) (ATP32)	Removal of H2O2, oxidation of toxic reductants, biosynthesis and degradation of lignin, suberization, auxin catabolism, response to environmental stresses such as wounding, pathogen attack and oxidative stress	UniProt (Reviewed)
Os10g0376400	Phosphate-induced protein 1 conserved region containing protein	Respond to abiotic stress (drought, cold, heat and salt)	Quan et al., 2018
Os10g0409400	beta subunit of polygalacturonase 1,	Abiotic stress response, Cell wall formation and abiotic stress response	Liu et al., 2013
Os10g0508700	Pectinesterase inhibitor domain containing protein	Control the activity of Pectin Methyl Esterase	Marzin et al., 2016
Os11g0300700	Protein kinase domain	Central components in plant responses to environmental stresses such as drought, high salinity, cold, and pathogen attack	Wang et al., 2020
**Downregulated in TN1 in all stresses**			
Os01g0311600	Sulfotransferase family protein	Respond to abiotic stress in rice; Asociated with drought, salt and ABA stress in chinese cabbaga	Chen et al., 2012; Jin et al., 2019
Os01g0823100	Alpha-expansin OsEXPA2	Involved in stem elongation; important for seed germination	Marowa et al., 2016; Huang et al., 2000
Os01g0871600	Peptide transporter PTR2-B	Various role in rice development mainly in grain filling and germination stages, Also upregulated upon drought and salt stresses	Ouyang et al., 2010
Os01g0953400	NB-ARC domain containing protein	Regulate the activity of R protein	Ooijen et al., 2008
Os02g0538400	Armadillo-like helical domain containing protein	Role in plant development and abiotic stress signalling	Sharma et al., 2014
Os02g0616300	Similar to Protein argonaute MEL1	Development of pre-meiotic germ cells and the progression of meiosis	Komiya et al., 2014; UniProt
Os03g0250200	TB2/DP1 and HVA22 related protein family protein	Inhibits gibberllin mediated PCD, involved in vesicular trafficking	Guo and Ho, 2008
Os04g0541100	Similar to Gt-2	Probable glycosyl transferase, required for cell wall synthesis	Cao et al., 2008
Os05g0469800	Pyruvate decarboxylase	Enzyme involved in alcohol fermentation, Also when overproduced enhance submergence tolerance in rice	Quimio et al., 2000
Os05g0477600	Alpha-expansin OsEXPA4	Internode elongation, Cell wall organisation in plants	Choi et al., 2003;
Os05g0488000	Peptidase C1A, papain family protein	Role in seed development and stress tolerance	Wang et al., 2018
Os05g0552400	Zinc finger, RING/FYVE/PHD-type domain containing protein	Involved in abiotic stress (salt and osmotic) tolerance in *Arabidiopsis*	Zang et al., 2016
Os06g0142300	Early nodulin 93 ENOD93 protein family protein	Candidate gene for brown plant hopper resistance in herbicide resistant rice; Required for somatic embryogenesis in oil palm	Wang et al., 2015; Chan et al., 2020
Os06g0179000	Glycoside hydrolase family 79, N-terminal protein	Response to biotic and abiotic stresses, defense against herbivores, activation of phytohormones, lignification, and cell wall remodelling	Opassiri et al., 2006
Os06g0549900	FAD linked oxidase, N-terminal domain containing protein	Required for the import and folding of small cysteine-containing proteins in the mitochondrial intermembrane space	UniProt
Os06g0610800	Peptidase A1 domain containing protein. Aspartic proteinase nepenthesin-1	Aspartic-type endopeptidase activity. Involved in biotic and abiotic stress responses	1.UniProt (reviewed); 2. Figueirido et al., 2021
Os07g0648000	Armadillo-like helical domain containing protein	Role in plant development and abiotic stress signalling	Sharma et al., 2014
Os08g0442400	BABY BOOM	Key regulators of plant cell totipotency. Induce somatic embryogenesis	Jha and Kumar, 2018; Khanday et al., 2020
Os08g0473900	1,4-alpha-D-glucan glucanohydrolase	Important for breakdown of endosperm starch during germination	UniProt; Guttikonda et al., 2020
Os08g0507100	Cytochrome P450 family protein	Function in development regulation and drought stress response	Wei and Chen, 2018
Os09g0241700	Homeodomain-like containing protein	Negative regulators in abiotic stress responses; Play role in reproductive development and abiotic stress signaling in rice	Bhattacharjee et al., 2016; Jain et al., 2008
Os09g0451400	ACC oxidase	Ethylene biosynthesis	Lee and Yoon, 2018
Os10g0109900	Major facilitator superfamily, general substrate transporter domain containing protein	Transport small compounds across biological membranes. Cadmium transporters in rice	Drew et al., 2021, Nino-Gonzalez et al., 2019; Yan et al., 2019
Os10g0556100	beta-expansin EXPB4	Internode elongation in rice	Lee et al., 2001
Os11g0212900	Serine/threonine protein kinase-related domain containing protein	Involved in abiotic stress (salt and osmotic) tolerance in *Arabidiopsis*	Zang et al., 2016
Os11g0508600	Sugar transporter	TAL effector-mediated susceptibility to bacterial pathogen (Xanthomonas)	Teper and Wang, 2021
Os11g0598300	NB-ARC domain containing protein	Regulate the activity of R protein	Ooijen et al., 2008
Os11g0672300	Protein kinase domain containing protein	central components in plant responses to environmental stresses such as drought, high salinity, cold, and pathogen attack	Wang et al., 2020
Os11g0675200	NB-ARC domain containing protein	Regulate the activity of R protein	Ooijen et al., 2008
Os11g0676200	NBS-LRR-like protein NBA2 (Fragment)	Component of R protein, Induce effector triggered immunity	DeYoung and Innes, 2006; Sagi et al., 2017
Os12g0569800	Heat shock protein 70	Involved in macromolecular translocation, carbohydrate metabolism, innate immunity, photosystem II repair and regulation of kinase activities	Wang et al., 2014
Os12g0637400	Purple acid phosphatase (EC 3.1.3.2)	Inorganic Phosphorous remobilization from senescing to non-senescing leaves and organic Phosphorous utilization	Gao et al., 2017

### Meta-analysis, narrowed down candidate genes for combined stress tolerance

To identify the key genes involved in combined stress, a meta-analysis was conducted using transcriptomic data from resistant and susceptible genotypes (our study) and microarray data from individual drought and pathogen infection from public domain (Additional file 4). The data was curated and analysed from both the approaches. Common and unique genes in individual and combined stress were identified which acts as candidate genes to develop multi-stress tolerant crops (Table 2, Additional file 5). In upregulated genes, under drought stress 230 genes were unique in BPT5204 and 79 genes were common in both microarray and RNA Seq data. Similarly 264 genes were unique in TN1 and 75 genes were common in microarray and RNA Seq data. This analysis identified, 14 unique genes that were commonly upregulated in BPT5204 and TN1, 5 genes were found to be common in both RNA Seq and microarray data. There are 22 unique genes and 6 commonly downregulated genes were identified (Table 2, Additional file 5).

**Table 2:** Differentially expressed genes from pathogen, drought and combined stress from RNA sequencing data generated from this study and microarray data from public domain

Drought	Upregulated genes			Downregulated genes		
	**Unique**	**Common**	**Microarray**	**Unique**	**Common**	**Microarray**
BPT 5204	230	79	6120	297	54	6046
TN1	264	75	6124	460	94	6006
*Common *^*a*^	14	5		22	6	
**Pathogen**						
BPT 5204	278	51	5041	432	72	3496
TN1	194	70	5022	374	39	3530
*Common*^*a*^	19	11		36	5	
**Drought + Pathogen**						
BPT 5204	394	53	2593	423	33	1930
TN1	487	108	2538	585	45	1918
***Common***^***a***^	**84**	**26**		**0**	**9**	

^a^ The list of these genes were given in additional file 5

In pathogen infection, 278 genes were uniquely upregulated in BPT5204 and 51 genes were common in microarray and RNA seq data. In TN1, 194 genes were unique and 70 genes were found upregulated in both microarray and RNA Seq data. Meta-anaylsis with RNA Seq and microarray revealed 19 unique genes between the genotypes and 11 common genes. In downregulated genes, 36 unique and 5 common genes were identified (Table 2, Additional file 5).

In combined stress, 394 genes were uniquely upregulated in BPT5204 and 53 genes were common in microarray and RNA Seq data. 487 genes were upregulated in TN1 and 108 genes were common in microarray and RNA Seq data. From meta-analysis, 84 unique genes and 26 common upregulated genes were identified. Interestingly, no downregulated unique gene in the genotypes and 9 genes were common in both RNA Seq and microarray (Table 2, Additional file 5). Overall from our genotypes identified many genes and using meta-analysis, key important genes which may be more relevant for improving combined stresses were identified.

### Differential responses of translation associated genes

Translation associated genes were differentially regulated in combined stresses. Ribosomal protein encoding genes play an important role in both biotic and abiotic stress conditions. To study the responses of translational associated mechanisms during the combined stress in both BPT5204 and TN1, the transcripts encoding ribosomal proteins (RP) were filtered from RNA seq data. In drought condition, 19 genes were commonly upregulated in both genotypes and same number of genes were downregulated (Table 3, Additional file 6). The number of genes that were up and downregulated were more in TN1 compared to BPT5204 indicating the severity of stress on that genotype. In pathogen infection, 26 and 34 genes were up and downregulated, respectively. Interestingly, more no. of genes were upregulated in BPT5204. In combined stress, 29 genes were upregulated and 27 genes were downregulated in both genotypes. The no. of genes upregulated in BPT5204 is less than TN1 genotype.

**Table 3:** Differential expression of ribosomal protein encoding genes from RNA sequencing data

**Drought**	**Upregulated**		**Downregulated**	
	**Unique**	**Common**	**Unique**	**Common**
BPT5204	12	19	7	19
TN1	37		11	
**Pathogen**				
BPT5204	22	26	35	34
TN1	7		27	
**Drought + Pathogen**				
BPT5204	16	29	27	27
TN1	22		26	

**Fig. 6:**
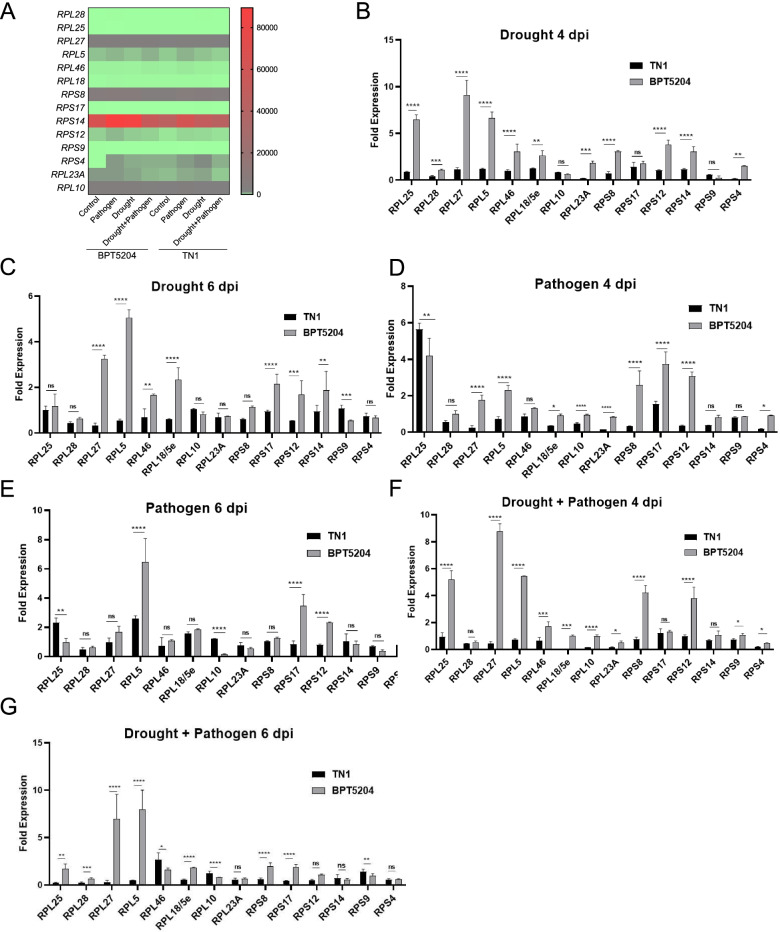
Response of ribosomal protein encoding genes in combined and individual pathogen and drought stress. **A** Multiple gene expression viewer showing differential response of ribosomal protein encoding genes from RNA sequencing data under pathogen, drought and combined stress in BPT5204 and TN1 genotypes. Average FPKM values from each stress was plotted using graphpad. (**B** and **C**) Expression of ribosomal protein encoding genes at 4 and 6 days after drought stress, (**D** and **E**) *Xoo* pathogen stress, and (**F** and **G**) combined stress. The leaf samples were collected after 4 and 6 dpi and total RNA was isolated. The cDNA was prepared and used for qRT-PCR analysis. Values are means ± SE with three biological replicates. Significant differences were determined at *p* < 0.0001 (estimated by one-way ANOVA using Tukey’s HSD analysis

To validate a few RP encoding genes using qRT-PCR, tissues were collected from 4 and 6 dpi. The expression of *RPL28, L25, L27, L46, L5e/L18, L5, L23, L10, RPS8, S18, S17, S14, S12* and *S4* genes were assessed in the RNA seq from both genotypes showing differential expression profile (Fig. 6A). The expression analysis study of RP encoding genes in drought, at 4 d showed upregulation in BPT5204. *RPL25, RPL27, RPL5, RPL46, RPS12* and *RPS14* were upregulated > 2 fold at 4 days (Fig. 6B) and at 6 days varied expression levels observed. However, *RPL27* and *RPL5* has maintained > 2 fold expression in BPT5204 (Fig. 6C). In pathogen infected condition at 4 dpi, all the RP encoding genes were upregulated in BPT5204 except *RPL25*. Expression of *RPS8, RPS17* and *RPS12* was upregulated > 2 fold (Fig. 6D). At 6 dpi, *RPL5* and *RPS17* transcript levels were >2 fold in BPT5204 compared to TN1 (Fig. 6E). In combined stress of drought and pathogen, the levels of RP encoding genes were significantly upregulated than individual stresses in resistant BPT5204 genotype. In combined stress, at 4 dpi transcripts of *RPL25, RPL27, RPL5, RPS8*, and *RPS12* were > 4 fold upregulated in BPT5204 compared to TN1. Other genes were also upregulated in resistant BPT5204 genotype (Fig. 6F). At 6 dpi transcripts of *RPL27* and *RPL5* were maintained > 8 fold in BPT5204 compared to TN1. The transcripts of other genes were maintained at higher levels in BPT5204 than TN1 genotype (Fig. 6G).

## Discussion

Rice is affected by plethora of stresses like drought, bacterial blight caused by *Xoo*, that are major constraints causing substantial crop loss. During drought stress in rice plants, reduction in fresh and dry biomass, plant height, tiller number, panicle number lead to crop loss. Many QTLs for drought stress tolerance are introgressed into elite varieties to improve crop yields. Similarly, many QTLs against *Xoo* has been identified to improve resistance to bacterial leaf blight (BB) disease. Efforts has been made to introgress BB resistant *Xa* genes with drought QTLs. Rice genotypes having *Xa4/qDTY2.2* + *qDTY4.1* showed improved resistance for combined drought stress and *Xoo* infection. Many genes have been identified and characterized for individual stresses, however, tolerance traits for abiotic and biotic stresses are multigenic in nature. From this context, developing durable climate resilient crops are in demand. To develop durable resistant crops, the candidate genes are prerequisite for improving combined stress tolerance.

Studying the simultaneous stress occurrence / combined stress experiment on plants in laboratory conditions are challenging due to the lack of stress imposition methods. In rice, transcriptome data was developed in combined drought and pathogen infection when plants reached 20% FC [18]. At 20% FC in drought condition, pathogens do not infect rice to cause disease, because of higher ROS that is cytotoxic. However, during severe drought stress, tissue water status reduces which inhibit bacterial growth in intracellular spaces [21]. To overcome this, we infected the plants with *Xoo* at mild drought stress (80% FC). At mild drought stress condition, water status of leaf reduces moderately and when pathogens are challenged, they can cause severe infection as observed in our study. When *Xoo* was infected at 80% FC, pathogen multiplied at higher rate causing more lesion length as evident in combined stress in both genotypes. At 80% FC, tissue water status does not reduce drastically which is favouring pathogen to infect the rice plants efficiently. The infection of *Xoo* on rice plants by leaf clipping method does not add additional water supply as in Arabidopsis and do not change relative water content of leaf. In this condition, pathogen infection is increased in combined stress. In resistant genotype BPT5204, higher ROS at 60% FC decreased the bacterial growth.

BPT5204 was introgressed with *Xa5, Xa13* and *Xa2*1 to improve resistance against *Xoo* [22]. To identify common and unique genes which can be involved in resistance under combined stress, comparative transcriptome data from resistant genotype with sensitive genotype and public data sets may provide relevant genes [23, 24]. A meta-analysis can integrate multiple transcriptomic data from different set of experiments, which provide an option to identify overlapping genes between drought and BB infection, to improve multi-stress tolerant plants using relevant candidate genes.

The RNA Sequencing data from BPT5204 and TN1 showed upregulation of many *peroxidases*, *cinnamoyl-CoA* genes*, starch and sugar metabolism* genes that are involved in phenylpropanoid biosynthesis pathway in both the genotypes. In combined stress, genes encoding t*hiolase-like protein, WRKY70, fatty acid elongase 1, Calcium dependent kinases* were upregulated. Many kinases were upregulated that in turn triggered many hormone signalling genes. Many candidate genes were identified for combined stress tolerance from these genotype. Meta-analysis identified 110 genes in combined stress, which were upregulated from different studies (Table 2). Interestingly, many of these genes were characterized for individual stresses (Table 1). In combined stress, kinases like *serine kinase (Os05g0466900, Os12g0454800)* were upregulated, similarly *leucine-rich repeat genes (Os01g0162300, Os01g0162500), ras-related protein (Os01g0750000), MTN3 (Os01g0606000), phosphofructokinase (Os06g0326400), cyclin-dependent kinase inhibitor (Os09g0459900)* genes were upregulated. Overexpression of *serine/threonine kinases* showed improved abiotic stress tolerance in Arabidopsis [25]. The role of *Os12g0454800* in cytokinin signalling has been deciphered [26]. A leucine-rich protein *Os02g0536300* involved in sensing PAMP responses to trigger the plant immunity [27]. Like that many serine threonine protein kinases have been characterized which are involved in improving the stress conditions to mediate cellular responses. In individual and common drought stress,12 transcription factors like *Zinc finger RING-type domain, HOX29, heat shock transcription factor 31, bZIP (Os02g0578500),* transcription activator for tolerance to drought, high-salt and cold stresses *(Os09g0522200), elongation factor, NAC, NAM, Class-B HSF (Os08g0546800), HSF29, HOX22, Homeodomain-leucine zipper (HD-Zip)* genes were upregulated. AP2 domain containing protein *RAP2.6* was downregulated. Transcription factors like *NAC, WRKY, bHLH, bZIP* were induced upon drought stress and bacterial blight infection. TFs regulate many downstream target genes [28–32]. *WRKY45* showed broad spectrum resistance and acts as a negative regulator for pathogen, salt, cold and drought stress is upregulated in combined stress [33, 34]. *WRKY11* acts as a positive regulator of defence response against *Xoo* and drought tolerance is upregulated in combined stress [35]. Genes encoding domains of unknown function (*DUF) 250, 868,761* were upregulated in combined stress. *DUF 810* improved drought and salt stress [36]. In combined stress, *ACC oxidase (Os09g0451400)* was upregulated and showed to involve in ethylene pathway indicating hormonal biosynthesis, play key role in combined stress tolerance [37].

Few *peptidases A1, aspartic proteinase nepenthesin-1 (Os06g0610800), serine carboxypeptidase 1 (Os04g0176400), peptidase S8 (Os10g0524600), and M50* family genes *(Os03g0729000)* were upregulated in combined stress. *Peptidase A1* encoding aspartic type endopeptidase activity showed improved biotic and abiotic stress [38]. Similarly *peptidase C1A* showed to play a role in seed development and improved stress tolerance [39]. Many *UDP-glucuronosyl and UDP-glucosyltransferase* were upregulated in combined stress [40]. Few chaperons like *Cpn60, copper chaperone homolog CCH, HSPs, dehydrin*s were upregulated in combined stress and involved in improving biotic and abiotic stress tolerance in many plants [41]. The DEGs identified in combined stress have potential to improve multiple stress tolerance in rice.

Translation associated genes are differentially expressed in combined stress. Ribosomal proteins are a class of highly conserved proteins across the living system involved in translation mechanisms. Among them, many are considered to have an important role during growth, development and stress condition in plants [42]. Many omics reports, represent genes associated with translation mechanism that are differentially regulated in individual as well as combined stress [7]. Recent studies, have shown extra-ribosomal function of ribosome encoding genes. There are 29 ribosomal protein encoding genes that were upregulated in both the genotypes in combined stress. Subsequent, validation of these genes confirmed upregulation in BPT5204 in combined stress, however, response of these genes varied from individual drought and pathogen infection. *RPL10* was upregulated at early time points in resistant genotypes, however, in TN1 upregulation was at 6 dpi indicating mechanism of early sensing of stress in tolerant variety compared to sensitive genotype. Mutation/silencing of RPL10 in *Arabidopsis*, *Nicotiana benthamiana* showed susceptible phenotype and weak ABA response [43, 44].

Genome wide expression analysis of rice in drought and *Xoo* showed upregulation of *RPL12, L28, L38, L36, L44 and L51*. In combined stress, expression of *RPL28, L25, L27, L5, L46, L18* was upregulated, similarly *RPS14, S12, S9, S4* were upregulated in resistant genotypes, *RPS6, RPS9* and *RPS10* were responsive to biotic stress [45]. *RPL10* play a vital role during both viral and bacterial infection acting as a positive and negative regulator [46]. Virus-induced gene silencing of *RPS12* and *RPS19* in *N*. *benthamiana* showed compromised non-host disease resistance [47]. Mutated *rpl23* plants showed to have impaired growth and developmental abnormalities [48]. *rpl27* mutant plants showed impaired shoot development and seed setting [49]. Our study shows elevated levels of *RPL23A* and *RPL27* during drought and combined stress. *RPS14* showed higher transcript levels upon hormonal treatment [50] and pathogen infection [51]. *RPS14* also showed a similar pattern of upregulation during all stress conditions. These evidences clearly show their extra-ribosomal functions in regulating stress adaptation. These ribosomal proteins play critical role in both transcriptional and translational mechanisms and differential expression of these genes indicate their potential role in improving multi-stress tolerance. More detailed studies are required to unravel these genes potential in stress adaptation.

## Conclusion

Developing climate resilient crops are in demand to supply the food for growing population. The relevant genes to improve multi-stress tolerance can be identified from plants which are simultaneously exposed to different combination of stresses. We have optimized combination of drought and bacterial infection process in rice and developed transcriptome information from contrasting genotypes. We demonstrate that, the role of many candidate genes which showed to improved stress tolerance for both drought and pathogen infection. Many of the genes were functionally validated by different research groups. These genes could be used to develop durable multi-stress tolerant crops in changing climatic conditions. Many candidate genes can be used for introgression in elite genotype background and also can be targeted for genetic manipulation using gene editing approaches.

## Material and methods

### Plant materials and growth conditions

Rice seeds of BPT5204 and TN1 genotypes collected from National Seed Project (NSP), University of Agricultural Sciences, GKVK, Bengaluru were used for this study. BPT5204 rice genotype is resistant to bacterial blight and is highly cultivated and TN1 genotype is susceptible for drought and bacterial blight. Four different sets i.e. control, drought, pathogen and combined stress were imposed. Seeds were soaked in water for O/N followed by germination on wet filter paper in Petri-plate. The germinated seedlings were transferred to individual pots, kept in green house condition (28 °C, 60% relative humidity and 16 h light / 8 h dark condition) and maintained. 45—day-old plants were used for imposing drought, pathogen and combined stress.

### *Xanthomonas oryzae pv. oryzae (Xoo)* inoculation and leaf sampling

*Xoo* culture was grown in nutrient broth (NB) medium (1% polypeptone, 0.5% yeast extract, 1% sucrose, pH 6.8) at 28˚C for 48 h. The *Xoo* inoculum was prepared by suspending the bacterial cells in 10 mM MES buffer. Leaves of 45-day-old plants were infected with 0.5 × 10^8^ CFU/mL of *Xoo* inoculum by leaf clipping method [52]. Bacterial disease symptoms were observed at 4 days of post infection (dpi), 6 dpi, 8 dpi, 10 dpi, 12 dpi and 14 dpi and bacterial growth was measured from the infected leaves.

### Drought and combined stress imposition

For drought stress, 45-days-old rice plants were exposed to gradual reduction in soil moisture content till 60% field capacity (FC) and further maintained along with the respective control. The samples were collected after plants reaching 60% FC for RNA sequencing. For combined stress imposition, 45-days-old rice plants were exposed to moisture stress by gradual reduction in watering till they reach 80% FC, then plants were infected with *Xoo* (5*10^8^ CFU/mL) and further maintained till 60% FC. Bacterial disease symptoms and CFU was measured at 6, 8, 10 and 12 dpi. After 51 days, tissues from minimum 5 plants were pooled in each sample, three biological replicates were collected from each pathogen, drought and combined stress exposed plants along with their respective controls.

### Determination of hydrogen peroxide (H_2_O_2_) by Diaminobenzidine (DAB) staining

Detached rice leaves were immersed in 1 mg/mL DAB (SRL- Sisco Research Laboratories, New Delhi, Cat no. 17076) solution at 3.8 pH. Leaves were infiltrated and kept in box for 5–6 h until brown precipitation was observed. Chlorophyll, was removed from the leaves with ethanol washing. Stained leaves were fixed in ethanol: acetic acid: glycerol (3:1:1) and photographs were taken. For quantification, stained leaves were ground and accumulation of formazan (reddish brown colour) was quantified by measuring the absorbance at 450 nm.

### Determination of superoxide anion radicals by using Nitroblue tetrazolium chloride (NBT)

Superoxide ion (O_2_^−^) react with NBT to form blue colour. Leaves were excised and kept in 0.1% (w/v) Nitro Blue Tetrazolium (NBT), 10 mM sodium azide and 50 mM potassium phosphate solution (pH 6.4). Leaves were vacuum infiltrated for 2–3 times until leaves were completely infiltrated. Further, leaves were kept in 10 mL of 0.1% NBT for 15 min. Chlorophyll was removed from leaves by washing with ethanol. Photographs were taken and quantification was done by measuring the absorbance at 560 nm.

### Cell membrane damage by Evan’s blue staining assay

Evans blue (Sigma-Aldrich, Cat no. E2129) solution was prepared in 0.1 M CaCl_2_ solution at pH 5.6. Tissues were dipped in Evan's blue solution for overnight and excess unbound dye washed with water. Images were taken under microscope. To quantify Evan's blue, dye was extracted in 1% SDS from the stained tissues and centrifuged for 5 min at room temperature to remove debris and elute dye into the supernatant. Optical density was measured at 600 nm and 1% SDS was used as blank. Concentration of Evans blue dye was estimated using standard curve method [53].

### RNA extraction and quantitative real-time PCR analysis

For RNA isolation from drought, pathogen and combined stress, plant samples were collected at 4 dpi and 6 dpi from both genotypes along with respective control. Samples were frozen in liquid nitrogen, crushed to powder and RNA was isolated using TRIzol reagent (Sigma-Aldrich, Cat no. T9424). Total 5 µg of RNA was then converted to cDNA using MMLV reverse transcriptase (ThermoFisher Scientific, Cat no. EP0451) with oligo dT primers. Specific primers for quantitative real-time PCR (qRT-PCR) were designed (Additional file 7). The qRT-PCR was performed using diluted cDNA and SYBR green (Takara Bio, Cat no. RR820A) on a Quant studio 6 Real Time PCR system (ABI-Quant studio 6 Real Time PCR system, ThermoFisher Scientific, Singapore). The expression data was collected and further processed to calculate 2^−ΔΔCT^ method [54]. Rice actin was used as internal control for normalization and three biological replicates were used for each gene.

### RNA sequencing and data analysis

For RNA sequencing analysis, infected leaf samples were collected at 4 dpi and frozen in liquid nitrogen. Since the disease progression was slow at 4 dpi and at 6 dpi the bacterial load is very high, plants trigger many transcriptional reprograming and to capture the differences we have collected the tissue at 4 dpi. Samples were collected in three biological replicates from both BPT5204 and TN1 genotypes. RNA sequencing was performed using Illumina HiSeq2500 platform from cDNA library by Theracues Innovations Pvt. Ltd., Bengaluru, India. The raw data was trimmed and low-quality reads were removed by the sickle trimming tool. The transcriptome analysis was performed using CLC Genomics Workbench v.12. The default parameters and analysis procedure followed as per CLC Genomics Workbench manual instructions. The control versus stress comparison [drought, pathogen and combined stresses (drought + pathogen)] was done in both genotypes and the IRGSP1.0 rice genome was considered as a reference for the analysis. The False Discovery Rate (FDR) ≤ 0.05, and log_2_FC ≥ 1.5 (for up-regulation), ≤ -1.5 (for down-regulation) rigorous filtering parameters were applied for the mining of differentially expressed genes (DEGs). The functional descriptions of the DEGs were retrieved from the Rice Annotation Project (RAPDB) database. The downstream analysis like pathway mapping, Gene ontology were carried out for DEGs using web-based tools KEGG mapper using mapping parameters mismatch cost = 2, insertion cost = 2, deletion cost = 3, length fraction = 0.8 and similarity fraction = 0.8. Transcripts Per Million (TPM) was used for the expression calculation. The agriGO (v2) (https://agrigo.rw/) analysis was performed using default settings.

### Meta-analysis

Meta-analysis was performed using RNA sequencing data of BPT5204 resistant and TN1 susceptible genotypes and microarray data curated from individual drought and pathogen infection from public domains (RiceMetasysA http://14.139.229.201/RiceMetaSys/ and RiceMetasysB http://14.139.229.201/RiceMetaSysB/**)*.***

## Supplementary Information

**Additional file 1. **Differentially expressed genes from contrasting genotypes identified in RNA sequencing data.

**Additional file 2. **Gene ontology of DEGs from BPT5204 and TN1.

**Additional file 3. **List of upregulated and downregulated genes having biological processes, molecular function and cellular components.

**Additional file 4. **List of DEGs from microarray data set.

**Additional file 5. **List of upregulated and downregulated genes in combined stress.

**Additional file 6. **DEGs of ribosomal protein encoding genes.

**Additional file 7. **List of primers used in this study

## Data Availability

All the data generated and analysed during this study are included in additional files. RNA sequencing datasets associated with this manuscript have been deposited on Gene Expression Omnibus (GSE197133) and can be accessed using the following link https://www.ncbi.nlm.nih.gov/geo/query/acc.cgi?acc=GSE197133. Go analysis—agriGO (v2) (https://agrigo.rw/); KEGG Mapper- https://www.genome.jp/kegg/mapper/ RiceMetasysA http://14.139.229.201/RiceMetaSys/ and RiceMetasysB http://14.139.229.201/RiceMetaSysB/ Rice Annotation Project (RAPDB) database- https://rapdb.dna.affrc.go.jp

## Abbreviations

*Xoo*: *Xanthomonas oryzae* Pv. *Oryzae*
TN1: Taichung native 1
BPT5204: Samba Mahsuri;
QTL: Quantative trait Loci
*Xa*: *Xanthomonas oryzae pv. oryzae resistant allele*
DEGs: Differentially expressed genes
FC: Field capacity
Dpi: Days after post infection
ROS: Reactive oxygen species
NBT: Nitroblue tetrazolium chloride
DAB: Diaminobenzidine
BDP: BPT5204 exposed to drought and Xoo- Combined stress
BD: BPT5204 exposed to drought
BP: BPT5204 exposed to pathogen
TDP: TN1 genotype exposed to drought and Xoo- Combined stress
TD: TN1exposed to drought
TP: TN1 exposed to pathogen
RNA seq: RNA sequencing
RP: Ribosomal proteins
qRT-PCR: Quantitative real time polymerase chain reaction
BB: Bacterial leaf blight BB
*qDTY2.2*: QTL for drought tolerance and yield
CFU: Colony forming unit
